# Characterisation of Patients with Systemic Lupus Erythematosus in Malta: A Population Based Cohort Cross-Sectional Study

**DOI:** 10.1155/2018/2385386

**Published:** 2018-09-30

**Authors:** Rosalie Magro, Andrew A. Borg

**Affiliations:** Rheumatology Department, Mater Dei Hospital, Msida, Malta

## Abstract

Systemic Lupus Erythematosus (SLE) is a multisystemic autoimmune disorder. The aim of this study was to characterise the SLE patients living in Malta in order to estimate the prevalence and incidence of SLE and characterise the clinical presentation as well as identify any unmet needs. 107 SLE patients who fulfilled SLICC classification criteria were identified. These were invited to participate in the study by means of an interview, blood and urine tests, and filling of the following questionnaires: Fatigue Severity Scale (FSS), visual analogue scale (VAS) for fatigue, Hospital Anxiety and Depression Scale (HADS), VAS for pain, Pittsburgh Sleep Quality Index (PSQI), and modified Health Assessment Questionnaire (mHAQ). The estimated prevalence of SLE in Malta is 29.3 patients per 100,000 and the estimated incidence is 1.48 per 100,000 per year. 93.5% of SLE patients were female, and the mean age at diagnosis was 33.1 years. 60.8% were overweight or obese and body mass index (BMI) had a significant positive correlation with daily dose of prednisolone (R=0.177, p=0.046). 20.7% and 3.3% had a moderate and high disease activity, respectively, as measured by SLEDAI-2K. Disease activity had a significant positive correlation with functional disability measured by mHAQ (R=0.417, p<0.001). 56.5% had an abnormal level of fatigue (FSS >3.7) and 57.6% had a high level of anxiety (HADS ≥8). This study has identified a number of unmet needs of SLE patients, including obesity, uncontrolled disease activity, fatigue, and anxiety.

## 1. Introduction

Systemic Lupus Erythematosus (SLE) is an autoimmune disorder having multiple manifestations including those involving the skin, musculoskeletal, renal, neurological, haematological, cardiovascular, and respiratory systems. It affects females nine times more frequently than males and has a reported prevalence of 20 to 50 per 100,000 people in Europe. The aetiology includes a combination of genetic, epigenetic, and environmental factors, such as UV light and infections, that lead to an irreversible break in immunological tolerance. SLE is associated with the presence of autoantibodies, including those that target double-stranded DNA (dsDNA) and extractable nuclear antigens [1].

In Malta, patients diagnosed with SLE are regularly followed up under the care of rheumatologists at the main general hospital, Mater Dei Hospital. Prior to this study, there was no local up-to-date register of SLE patients and a population based study on SLE had not been described; thus the prevalence and incidence of SLE in Malta was not known. Population based studies in other Mediterranean islands, namely, Sardinia and Crete, have noted a prevalence of SLE of 81 and 123.4 per 100,000, respectively [2, 3]. The incidence of SLE in Crete was reported as 7.4/100,000 per year [3].

The aim of this study was to characterise the patients with SLE living in Malta. This involved inviting all diagnosed SLE patients to participate in the study, thus enabling a population based study. The study aimed to characterise the patients in terms of disease characteristics including age of disease onset, organ involvement, autoantibodies present, disease activity, and damage. This would allow comparison with other populations of SLE patients, whose characteristics are already known. The study also looked into additional characteristics that are not disease-specific, including fatigue, sleep quality, depression, anxiety, functional disability, and serum vitamin D level. This would enable the identification of any unmet needs with regard to these aspects in this group of patients.

## 2. Method

The study consisted of a cross-sectional cohort study of SLE patients in Malta. All known cases of SLE over the age of 17 and who fulfil the SLICC classification criteria for SLE were invited to participate in the study [4]. Since a formal register of SLE patients was not available, cases of SLE were identified through various sources. These included (i) referral from all local Rheumatology consultants and doctors working within the Rheumatology Department and (ii) inviting patients who are members of local patient associations and SLE support groups. 129 patients were referred for inclusion in the study. Of these, 22 patients did not fulfil the SLICC classification criteria for SLE and were thus excluded from the study [4]. Approval to carry out the study was obtained from the University Research and Ethics Committee.

Demographic data and information on disease onset, comorbidities, autoantibody status, SLE organ involvement, and drug history was obtained from the medical notes for the 107 patients eligible for inclusion in the study. Out of these patients, 92 patients gave informed consent to participate further in the study by means of an interview and questionnaires and blood and urine tests. A face-to-face interview was conducted with each of the 92 participants and the following information was collected: smoking status, physical activity, use of sunscreen, body mass index (BMI), drug history (including calcium and vitamin D supplements), SLE disease activity index (SLEDAI 2K) score, and Systemic Lupus International Collaborating Clinics/American College of Rheumatology damage index (SDI). Fatigue, depression, pain, sleep quality, and disability have been assessed, respectively, by asking the patients to fill in the following questionnaires: Fatigue Severity Scale (FSS), visual analogue scale (VAS) for fatigue, Hospital Anxiety and Depression Scale (HADS), VAS for pain, Pittsburgh Sleep Quality Index (PSQI), and modified Health Assessment Questionnaire (mHAQ) [5–10]. The questionnaires were available in both English and Maltese; the questionnaires used depended on the patients' preference. The Maltese translation of the questionnaires used has already been validated [11, 12]. The following blood tests were taken: full blood count, thyroid function test, 25-hydroxyvitamin D, erythrocyte sedimentation rate (ESR), C-reactive protein (CRP), complement 3 (C3), complement 4 (C4), anti-double-stranded deoxyribonucleic acid (anti-dsDNA), renal function, random blood glucose, calcium, and albumin. A sample of urine was sent for urinalysis and microscopy and for protein creatinine ratio. The data was collected over a 9-month period from October 2016 to June 2017.

All the statistical calculations were done using the statistical software IBM SPSS statistics 24. The normality of continuous variables was established by means of the Kolmogorov–Smirnov test. Normally distributed variables were summarized using the mean and standard deviation. The median and range were used for nonnormally distributed variables. For univariate comparisons between two continuous variables, Pearson's or Spearman's coefficients for normal or nonnormal variables, respectively, were used. Comparisons of continuous variables between two groups were done using independent samples T test in the case of normal variables and Mann–Whitney U test in the case of nonnormal variables. The Chi Squared test was used to compare categorical variables between two groups.

## 3. Results

**Figure 1:**
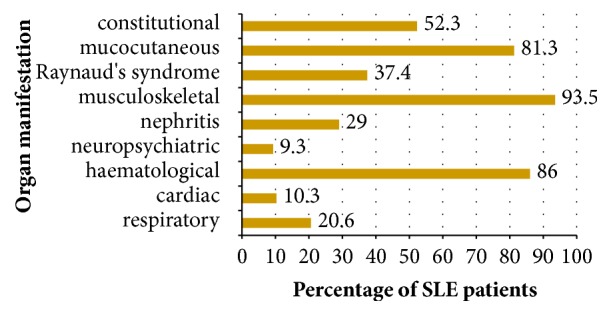
Chart showing percentage of SLE patients with various organ manifestations in SLE at any time during the course of the disease.

**Figure 2:**
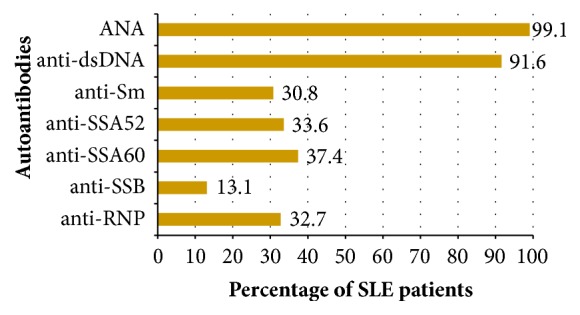
Chart showing percentage of SLE patients having positive autoantibodies.

93.5% of SLE patients studied were female. Out of the 107 patients, 104 were Caucasian (of which 95 were Maltese) and 3 were of Asian origin. The mean age was 46.2 years (range 19-79) and the median duration of SLE was 12 years (range 0-43 years). In the cohort studied, the age of SLE onset ranged from 3 to 71 years, with a mean age of onset of 33.1 years. Patients with a history of lupus nephritis and neuropsychiatric manifestations had a significantly lower mean age of disease onset (p=0.003, p=0.032). No significant difference in age of disease onset was noted for the other organ manifestations. Moreover, patients who were positive for anti-Sm and anti-RNP, had a significantly lower mean age of SLE onset (p=0.44, p=0.003). 79.4% of patients had at least one comorbidity. These included osteopaenia/osteoporosis in 30.8%, hypertension in 25.2%, hypercholesterolaemia (on treatment) in 11.2%, diabetes mellitus in 8.4%, fibromyalgia in 9.3%, antiphospholipid syndrome in 10.3%, Sjogren's syndrome in 3.7%, and rheumatoid arthritis in 2.8%. 58.9% were lifelong nonsmokers, 22.4% were ex-smokers, and 18.7% were smokers. The frequency of organ manifestations in SLE at any time during the course of the disease and autoantibody profile are depicted in Figures 1 and 2, respectively.

45.8% of SLE patients studied were receiving prednisolone at a dose ranging from 1.25mg to 15mg daily. 52.3% received calcium supplementation and 57.0% received vitamin D supplementation. 60.7% of SLE patients were receiving hydroxychloroquine, 20.6% were on azathioprine, 9.3% on methotrexate, and 8.4% on mycophenolate mofetil. One patient was receiving rituximab. Out of the 42 patients not receiving hydroxychloroquine, 22 patients (52.4%) were previously on it and had been stopped for several reasons, including side effects and disease remission. These 22 patients were previously on hydroxychloroquine for a mean of 1.6 years, ranging from few days up to 16 years. In the remaining 20 patients, it was documented that hydroxychloroquine was offered to 4 patients but it was refused by the patients. No documentation was found, with regard to discussion with the patient on hydroxychloroquine in the remaining 16 patients.

The characteristics of the 92 patients who gave consent to participate in the study by means of an interview, questionnaires, and blood and urine tests were compared with those of the 15 patients who did not. There was no significant difference in terms of age (p=0.736), disease duration (p=0.628), age of disease onset (p=0.174), gender (p=0.269), and ethnicity (p=0.328). This also applied to the antibody profile and comorbidities documented, with the exception of antiphospholipid syndrome (p=0.024). For the latter, 7.6% of patients who gave consent suffered from antiphospholipid syndrome, whereas this was the case in 26.7% of those who did not consent.

Out of the 92 patients who were studied in further detail, 3.3% had a family history of SLE in a first-degree relative and 5.4% in a second-degree relative. The median BMI was 26.5kg/m^2^ (range 17.7–53.5kg/m^2^); 1.1% were underweight (BMI < 25kg/m^2^), 38% had a normal BMI (BMI 18.5–25kg/m^2^), 31.5% were overweight (BMI 25–30kg/m^2^), and 29.3% were obese (BMI >30kg/m^2^). BMI had a significant positive correlation with daily dose of prednisolone (R=0.177, p=0.046). 50% of SLE patients claimed that they were using sunblock regularly at the time of the interview, at a frequency ranging from once a week to more than once a day. 41.3% claimed that they carried out regular exercise. The clinical characteristics of the cohort are summarized in Table 1.

23.9% of SLE patients were in remission (SLEDAI-2K 0), while 52.2% had a low disease activity (SLEDAI-2K 1-5) at the time of the interview. 20.7% and 3.3% had a moderate (SLEDAI-2K 6-10) and high (SLEDAI-2K 11-19) disease activity, respectively. With regard to damage, the median SDI is 1 (range 0–6). A significant positive correlation was noted between SDI and disease duration (R=0.355, p<0.001), and a significant negative correlation was noted between SLEDAI-2K and disease duration (R=-0.229, p=0.028). A significant positive correlation was noted between functional disability measured by mHAQ and SLEDAI (R=0.417, p<0.001). A significant negative correlation was noted between mHAQ and disease duration (R=-0.236, p=0.023). However no correlation was noted between SDI and mHAQ (R=-0.007, p=0.473).

Table 2 shows the results obtained for FSS, VAS fatigue, and pain and HADS, PSQI, and mHAQ. 56.5% were noted to have an abnormally high level of fatigue (FSS >3.7). 6.5% were noted to have depression (HADS D 11–21), 18.5% were borderline (HADS D 8–10), and 75% had a normal HADS D score (0–7). A higher frequency of anxiety was noted with 35.9% having anxiety (HADS A 11–21) and 21.7% were borderline (HADS A 8–10) [5]. 55.4% were noted to have poor sleep quality (PSQI >5) and 26.1% had an abnormal level of function (mHAQ >0.3) [8, 10].  Table 3 shows the results obtained for the blood and urine tests that were carried out. 2 patients had chronic kidney disease (CKD) stage 4 (estimated glomerular filtration rate (eGFR) 15–29) and 2 patients had CKD stage 5 (eGFR <15). 15.2% were found to have vitamin D deficiency (25-hydroxyvitamin D <20ng/ml) and 27.2% were vitamin D insufficient (25-hydroxyvitamin D 21–29ng/ml). Out of the remaining patients who had normal level of vitamin D, 75.5% were already receiving vitamin D supplementation. Overall, from the cohort of 92 patients, only 14.1% had a normal level of vitamin D and were not receiving vitamin D supplementation.

## 4. Conclusions

This is the first population based study on SLE to be carried out in Malta. Since the population of adults, over the age of 17 years, in Malta is estimated at 365,000, the prevalence of SLE in Malta is estimated to be 29.3 patients per 100,000 [13]. This has been calculated from the fact that, in this population based study, 107 patients over the age of 17, who fulfil the SLICC classification criteria for SLE, have been identified. The mean number of newly diagnosed cases of SLE in adults from 2012 to 2016 per year, in Malta, is 5.4. The estimated incidence is thus 1.48 patients per 100,000 per year. This is comparable to the reported prevalence and incidence rates in other European countries [14, 15]. Even though every effort was made to include all SLE patients in Malta, by not only including patients seen by all rheumatologists, but also by directly inviting patients in support groups, the estimated prevalence and incidence rates could be a slight underestimate. Moreover, since the estimated incidence rate took into account newly diagnosed cases over a five-year period, any fatalities of newly diagnosed cases in this period were not taken into account. As in other reported studies, the incidence and prevalence rates for men are approximately 1/10th those in women [14]. The disease onset is in the majority of cases between the ages of 20 and 40 years. The frequency of most organ manifestations at any time during the course of the disease is comparable to that from other cohorts, including the Euro-Lupus cohort, a ten-year prospective study carried out in Europe [16]. The frequency of neuropsychiatric manifestations was notably lower in our study, possibly because the data collected on organ manifestations was collected retrospectively from the medical notes and from the interview with the patient. Patients with a history of renal and neuropsychiatric manifestations had a younger age of onset of SLE. Other studies have also shown that renal and neurological manifestations are more common in SLE patients with a younger age of disease onset [17, 18].

60.8% were overweight or obese. This is slightly higher than the percentage of overweight and obese individuals in the Maltese general population (58.6%) [19]. This could be due to the effect of oral glucocorticoids, which were being taken by 45.8% of patients. In fact BMI had a significant positive correlation with daily dose of prednisolone (R=0.177, p=0.046). Current smokers are lower in the SLE cohort compared to the Maltese general population (25.9%), possibly as more SLE patients have stopped smoking due to the concurrent medical problems [19]. Comorbidities were frequent. Of note, 9.3% had also been diagnosed with fibromyalgia. This is higher than in the general population, where it is estimated around 2% [20]. This increased prevalence of fibromyalgia in SLE patients has also been reported in other studies [21]. A case-control study has suggested that anti-N-methyl-D-aspartate receptor antibodies are associated with the pathogenesis of fibromyalgia with SLE [22].

Antimalarials are strongly recommended in SLE to prevent flares and reduce the development of renal disease and chronic damage and for its steroid-sparing properties [23–28]. However, this study showed that only 60.7% of SLE patients were receiving hydroxychloroquine at the time of the study. 20.6% of the entire SLE cohort were not receiving hydroxychloroquine during the time of the study but had received hydroxychloroquine in the past. 18.7% were never on hydroxychloroquine, although this data is limited as it has been collected retrospectively by using the hospital medical records. Out of these patients, it was documented that hydroxychloroquine was offered to 4 patients.

15.0% were receiving 7.5mg daily or a higher dose of prednisolone. Moreover 3.3% had a high disease activity (SLEDAI >10). These patients require a tighter control of their disease activity and could be potential candidates for biological drugs such as belimumab. In the study it was also noted that the disease was more active in patients with shorter disease duration. The improvement of disease activity with time has also been shown in prospective studies [29, 30]. As expected, damage was higher in patients with a longer disease duration. This is also consistent with findings from prospective studies [29, 30]. Function as measured by mHAQ was influenced strongly by disease activity, but not by damage. Other studies showed a correlation of HAQ (Health Assessment Questionnaire) with both SLEDAI and SDI [31, 32]. In our study a relationship between health statuses measured by mHAQ was not significantly correlated to damage, possibly due to the limited size of our cohort and also since mHAQ was used, as opposed to HAQ.

Fatigue has been described as the most prevalent symptom in SLE, as it is present in up to 90% of patients [33]; it is considered to be the most disabling symptom in around half of the patients [5]. Fatigue was highly prevalent in the SLE cohort described in this study. The mean FSS in normal healthy adults is 2.3 [5]; in the SLE cohort this was 4.02. A high prevalence of anxiety was noted, with 35.9% having a HADS score above 10. This is in concordance with results from other studies, including a meta-analysis [34]. Vitamin D deficiency (serum 25-hydroxyvitamin D concentrations ≤20 ng/mL) is common; its prevalence in adults in Europe ranges from 34% to 67% [35]. Vitamin D deficiency is more prevalent among SLE patients, possibly due to sun avoidance and renal disease [36]. A high frequency of vitamin D deficiency and insufficiency was noted, with only 14.1% of patients not requiring vitamin D supplementation.

In conclusion, this study has defined multiple disease-specific and other characteristics of SLE patients in Malta, facilitating future planning in the management of this condition. Of note, it has identified a number of unmet needs that need to be addressed. These include the high frequency of obesity, fatigue, anxiety, and poor sleep quality. Other unmet needs include vitamin D deficiency and uncontrolled disease activity.

## Figures and Tables

**Table 1:** Clinical characteristics of the cohort.

**Characteristics**	**Values**
**Age, mean (S.D.) years**	46.2 (13.9)
**Female sex, n/N (**%**)**	100/107 (93.5)
**Caucasian race, n/N (**%**)**	104/107 (97.2)
**Disease duration, median (range) years**	12 (0-43)
**Age of SLE onset, mean (S.D.) years**	33.1 (13.3)
**BMI, median (range) kg/ m** ^**2**^	26.5 (17.7-53.5)
**Current smoker, n/N (**%**)**	20/107 (18.7)
**Current use of sunscreen, n/N (**%**)**	46/92 (50)
**Regular exercise, n/N (**%**)**	38/92 (41.3)
**Family history of SLE in first degree relative, n/N (**%**)**	3/92 (3.3)
**Any co-morbidity, n/N (**%**)**	85/107 (79.4)
**Osteopaenia/osteoporosis, n/N (**%**)**	33/107 (30.8)
**Hypertension, n/N (**%**)**	27/107 (25.2)
**Hypercholesterolaemia on treatment, n/N (**%**)**	12/107 (11.2)
**Diabetes mellitus, n/N (**%**)**	9/107 (8.4)
**Fibromyalgia, n/N (**%**)**	10/107 (9.3)
**Anti-phospholipid syndrome, n/N (**%**)**	11/107 (10.3)
**Sjogren's syndrome, n/N (**%**)**	4/107 (3.7)
**Rheumatoid arthritis, n/N (**%**)**	3/107 (2.8)
**Current prednisolone, n/N (**%**)**	49/107 (45.8)
**Prednisolone dose, median (range) mg/day**	5.00 (1.25-15.00)
**Current hydroxychloroquine, n/N (**%**)**	65/107 (60.7)
**Current azathioprine, n/N (**%**)**	22/107 (20.6)
**Current methotrexate, n/N (**%**)**	10/107 (9.3)
**Current mycophenolate, n/N (**%**)**	9/107 (8.4)
**Current rituximab, n/N (**%**)**	1/107 (0.9)
**Current calcium supplementation, n/N (**%**)**	56/107 (52.3)
**Current vitamin D supplementation, n/N (**%**)**	61/107 (57.0)
**SLE disease activity index 2K (SLEDAI 2K), median (range)**	4 (0-12)
**Systemic Lupus International Collaborating Clinics/American College of Rheumatology damage index (SDI), median (range)**	1 (0-6)

**Table 2:** Table showing results obtained for the questionnaires: Fatigue Severity Scale (FSS), visual analogue scale (VAS) for fatigue, VAS for pain, Hospital Anxiety and Depression Scale depression (HADS D), Hospital Anxiety and Depression Scale anxiety (HADS A), Pittsburgh Sleep Quality Index (PSQI), and modified Health Assessment Questionnaire (mHAQ).

**Characteristics**	**Values**
**FSS, median (range)**	4.17 (1-6.79)
**VAS Fatigue, median (range)**	5.00 (0-9)
**VAS Pain, median (range)**	4.00 (0-8)
**HADS depression, median (range)**	5.00 (0-13)
**HADS anxiety, mean (S.D.)**	8.48 (4.32)
**PSQI, median (range)**	6 (0-18)
**mHAQ, median (range)**	0.125 (0-2)

**Table 3:** Table showing results obtained for the blood and urine investigations carried out. Normal values have been included in square brackets. (eGFR: estimated glomerular filtration rate, CRP: C reactive protein, ESR: erythrocyte sedimentation rate, C3: complement 3, C4: complement 4, and anti-dsDNA: anti-double stranded deoxyribonucleic acid).

**Investigation**	**Value**
**Haemoglobin, mean (S.D.) ** *[12.0-15.5 g/dL]*	13.07 (1.60)
**Estimated glomerular filtration rate (eGFR), median (range) ** *[>60 mls/min/1.73m* ^*2*^ *]*	97.0 (5-155)
**Urine protein creatinine ratio, median (range) ** *[0-150 mg/g]*	96.9 (34.2-1583.4)
**25-hydroxyvitamin D, mean (S.D.) ** *[30-100 ng/mL]*	30.75 (9.53)
**Corrected calcium, median (range) ** *[2.05-2.60 mmol/l]*	2.30 (2.1-2.63)
**CRP, median (range) ** *[0-5 mg/L]*	1.65 (0.1-58)
**ESR, median (range) ** *[18-22 mm 1st hr]*	21 (2-114)
**C3, mean (S.D.) ** *[900-1800 mg/l]*	1010.5 (244.6)
**C4, mean (S.D.) ** *[100-400 mg/l]*	232.07 (108.8)
**Anti-dsDNA, median (range) ** *[0.0-100 IU/mL]*	92.9 (10-800)

## Data Availability

The descriptive data used to support the findings of this study are included within the article.
